# Interfacial properties of SiC_f_/SiC minicomposites with a scheelite coating

**DOI:** 10.1038/s41598-022-26626-9

**Published:** 2022-12-19

**Authors:** Na Ni, Binbin Wu, Yinchun Shi, Xiaohui Fan, Chuanwei Li

**Affiliations:** 1 School of Mechanical Engineering, Shanghai Jiao Tong University, Shanghai, 200240 China; 2 School of Material Science and Engineering, Shanghai Jiao Tong University, Shanghai, 200240 China

**Keywords:** Structural materials, Ceramics, Composites, Mechanical properties, Aerospace engineering

## Abstract

Unidirectional SiC_f_/SiC minicomposite with a scheelite (CaWO_4_) interphase coating was fabricated through the precursor infiltration and pyrolysis method. Fractography of the SiC_f_/SiC minicomposites indicated that weak fiber/matrix bonding can be provided by the CaWO_4_ interphase. Furthermore, interfacial debonding stress of SiC_f_/CaWO_4_/SiC minicomposite was evaluated through the fiber push-out test, and estimated to be 80.7 ± 4.6 MPa. In-situ tensile SEM observation of SiC_f_/CaWO_4_/SiC minicomposite after oxidation at 1000–1100 °C was carried out, and thermal compatibility between CaWO_4_ interphase coating and SiC fiber or matrix after heat treatment at 1300 °C was investigated.

## Introduction

Incorporation of reinforcing fibers into a brittle ceramic matrix provides a degree of pseudo-ductility to ceramic matrix composites (CMCs), typically the SiC fiber-reinforced SiC matrix composite (SiC_f_/SiC), preventing catastrophic failure by several mechanisms, such as fiber debonding, fiber sliding and crack bridging. SiC_f_/SiC composites are considered to be promising materials durable for severe environment applications such as super-sonic transport, space planes and fusion reactors^1–3^. The deviation of matrix cracks within the fiber-matrix interphase zone is realized and controlled by the deposition of a coating layer of a compliant material on the fibers prior to matrix fabrication^4–6^. The most effective interphase coating is considered to be PyC or BN coating^7–9^. However, the application of SiC_f_/SiC composites is limited by degradation of mechanical properties in oxidizing environments, due to oxidation of the PyC or BN coating at the fiber/matrix interface, particularly at intermediate temperatures^4, 10^.

Alternative oxidation-resistant interphase materials such as rare-earth orthophosphate monazite (LaPO_4_) have been considered as oxidation-resistant fiber-matrix interphases for CMCs due to the layered crystal structure and plastic deformation possibility. Studies on the LaPO_4_ interphase showed that it could be deformed under mechanical stresses at low temperatures by twinning and dislocation^11–13^. However, it was found to be thermodynamically incompatible with SiC^14, 15^. Both compatibility and oxidation resistance are important for the interphase coating to ensure the long period application of CMCs in air. Shanmugham et al. showed that a mullite interphase in SiC_f_/SiC composites deflected cracks even after exposure in air at 1000 °C for 24 h^16^. Lee et al. demonstrated the use of multilayer SiO_2_/ZrO_2_/SiO_2_ oxide coatings for SiC/SiC composites. Composite strength and crack deflection were retained after oxidation in air at 960 °C for 10 h^17^. Scheelite (CaWO_4_) has a layered structure consisting of (WO_4_) tetrahedra and eight-coordinated Ca sites and cleavage of its crystal was reported on (101) planes. The layered crystal structure and plastic deformation possibility of scheelite (CaWO_4_) make it another potential interphase material for SiC_f_/SiC composites^18–22^. So far, the fabrication of scheelite materials as the interphase coating in SiC_f_/SiC composite and the investigation on its effect has not been explored.

In this work, unidirectional SiC_f_/CaWO_4_/SiC minicomposite was prepared through the precursor infiltration and pyrolysis (PIP) method. The effectiveness of the CaWO_4_ interphase coating was investigated by comparing the fractography of SiC_f_/SiC composites with and without CaWO_4_ interphase coating. The interfacial property of the composites was quantitatively characterized by fiber push-out tests. The SiC_f_/CaWO_4_/SiC composites were oxidized at 1000–1100 °C to evaluate the oxidation resistance of the CaWO_4_ interphase coating in oxygen-rich environments. In addition, SiC_f_/CaWO_4_/SiC composites were heat-treated at 1300 °C to investigate the compatibility between CaWO_4_ interphase coating and SiC fiber or matrix.

## Methods

### Preparation of CaWO_4_ interphase coating

Continuous CaWO_4_ interphase coatings were prepared on KD-II SiC fiber (provided by NUDT, China) through the sol–gel method. The property of the fiber can be found in the reference^23^. The detailed coating process was described in our previous work^19, 20^. The optimized firing temperature of 900℃ and atmosphere in Ar were used to avoid the fiber strength degradation during the coating process. The suitable coating thickness was obtained by repeating the coating preparation process for 6 cycles.

### Preparation of SiC_f_/SiC minicomposites

**Figure 1:**
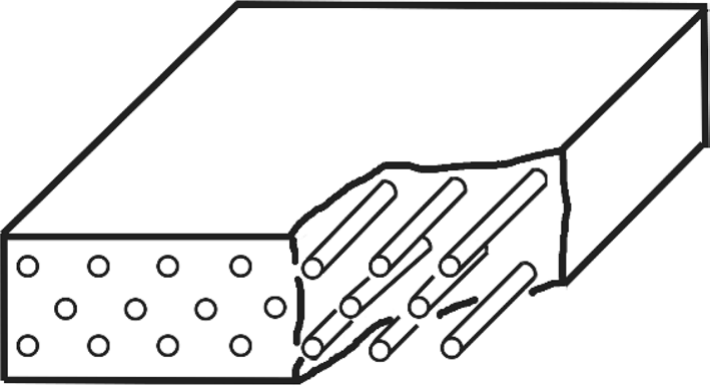
Schematic structure of the unidirectional SiC_f_/SiC minicomposite.

Unidirectional SiC_f_/SiC minicomposites with and without CaWO_4_ interphase coating were prepared through the polymer infiltration and pyrolysis (PIP) method. The schematic structure of the minicomposite is demonstrated in Fig. 1.

**Figure 2:**
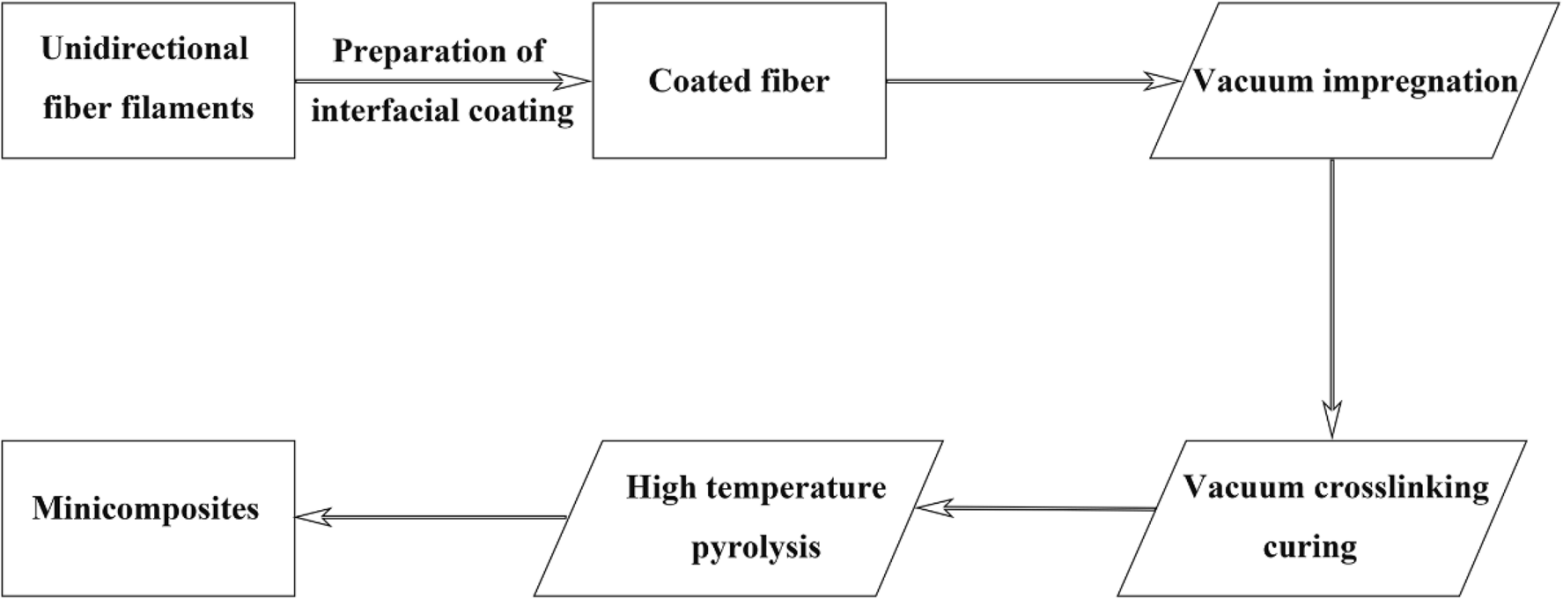
Preparation flow chart of SiC_f_/SiC minicomposites.

The flow chart for the preparation of SiC_f_/SiC minicomposites is shown in Fig. 2. The PIP process utilized polycarbosilane (PCS) to form SiC matrix on pyrolysis at 1100 °C in Ar^24^. The ceramic yield of PCS is about 81–85%, and its crystallization temperature is about 1000 °C. The choice of pyrolysis temperature was based on the balance of maximum matrix densification and minimum fiber degradation. For the impregnation and crosslinking process, firstly, SiC fibers were immersed into the slender crucible which contains PCS. Then, the crucible was transferred to the vacuum drying oven to complete the vacuum impregnation process, where continuous pumping can ensure more sufficient impregnation. After pumping for 2 h, the vacuum drying oven was slowly heated up to 130 °C to complete the vacuum crosslinking curing process. Apart from that, the SiC nanoparticles (40 nm) with a volume fraction of 30–40% were filled in PCS to reduce shrinkage cracking of the matrix during pyrolysis. The infiltration and pyrolysis process was repeated 7 times to improve the density of the minicomposites.

### Characterization

The composition of CaWO_4_ coated SiC fiber tows were examined by X-ray diffractometry (XRD, Ultimo IV, Riau, Japan). Morphology of coated SiC fibers and SiC_f_/SiC minicomposites was investigated by scanning electron microscopy (SEM, Inspect F50, FEI) equipped with energy dispersive spectroscopy (EDS). The porosity and density of SiC_f_/SiC minicomposites were measured through Archimedes' method of drainage.

**Figure 3:**
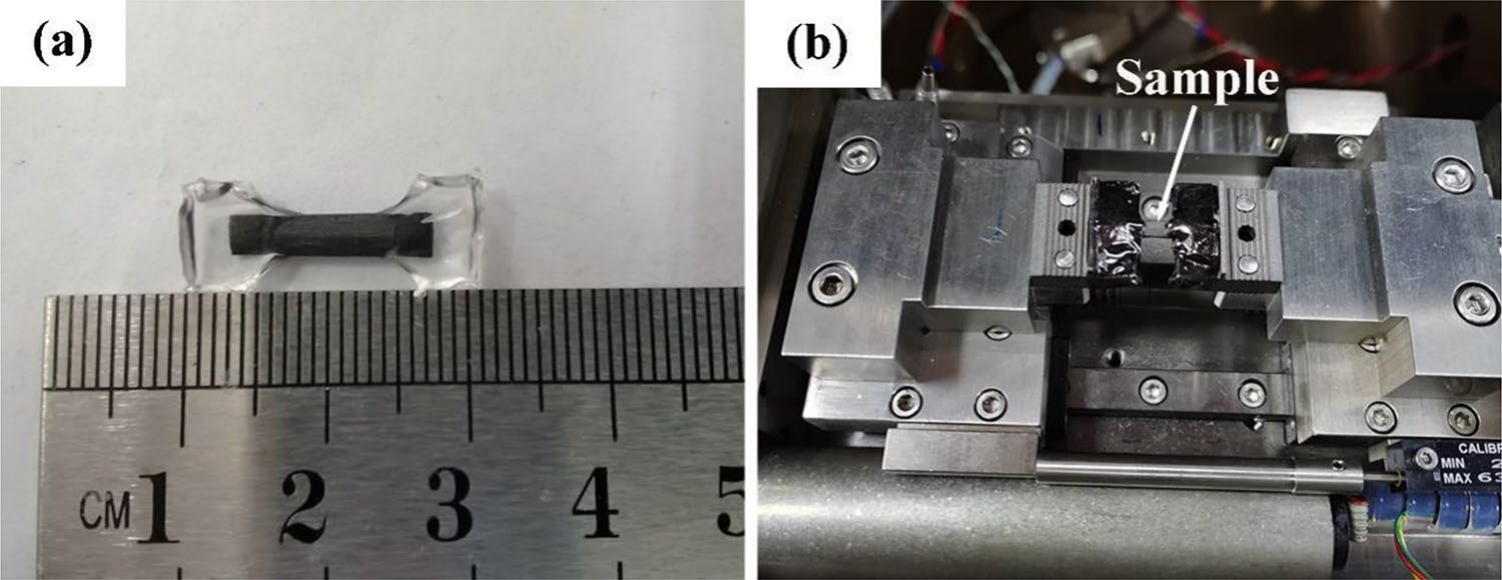
In-situ tensile SEM observation of SiC_f_/SiC minicomposite: (**a**) sample and (**b**) tensile platform.

In-situ SEM observation during the tensile tests of SiCf/SiC composites was performed using an SEM (Lyra3 GMU, Tescan, Czech Republic) in combination with an in-situ tensile stage (MTEST5000W, GATAN, UK) to investigate the fracture behavior of the composites. The composite specimens used for in-situ tests were approximately 1 × 2 × 15 mm in size, with their ends embedded in epoxy resin, and the area used for in-situ observation was approximately 2 × 4 mm. The samples were first gold sprayed (10 mA, 15 s, gold film thickness of approximately 2 nm) to increase the electrical conductivity before being placed in the SEM, and then the specimens were fixed on the tensile stage fixture (Fig. 3). In the in-situ tensile test, the stage used a displacement control mode, i.e., the motor loading rate was 0.1 mm/min until fracture. SEM imaging wad carried out using a backscattered electron detector with a working distance of 30 mm, a voltage of 10 kV, and beam current of 1 nA. An automatic image acquisition module with a single image acquisition time of about 3.2 s and an interval of 0 s was employed. The magnification chosen for in-situ observation was small, which can ensure that the sample was always within the field of view during the tensile process. After the sample fractured, the SEM still acquired several photographs to ensure that the whole process of stretching can be observed.

Single fiber push-out tests were carried out to quantitatively characterize the interfacial property of SiC_f_/SiC minicomposites. The minicomposites were first ground and polished to 100–300 μm, and then the slice was fixed to the graphite flake with a groove. The thickness of the composite has been reported to not affect the estimation of the interfacial debonding stress^25^. Finally, Agilent Technologies G200 nano indention was utilized to push the single fiber in the composites. The maximum load is 1000 mN.

To evaluate the potential performance of the CaWO_4_ interphase in high-temperature oxidizing environment, the SiC_f_/CaWO_4_/SiC minicomposites were oxidized at 1000–1100 °C in air for 10 h and 50 h in the muffle furnace (SXL-1400). Furthermore, the composites were heat-treated at higher temperatures of 1200–1300 °C for 10 h and 50 h in air to investigate the compatibility between the CaWO_4_ coating and SiC.

## Results and discussion

### Characterization of CaWO_4_ coated fibers and minicomposites

**Figure 4:**
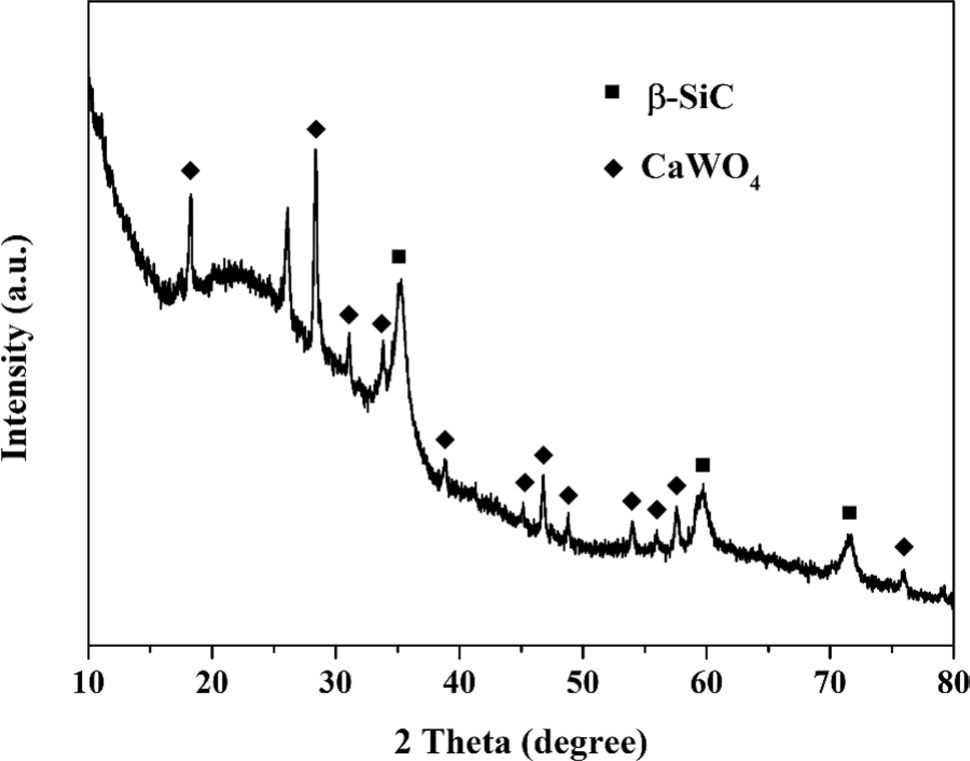
XRD pattern of CaWO_4_ coated SiC fiber.

According to the XRD analysis of CaWO_4_ coated SiC fiber shown in Fig. 4, only CaWO_4_ was identified besides the β-SiC phase originating from the underlying SiC fiber, suggesting that the prepared interphase coating is pure CaWO_4_.

**Figure 5:**
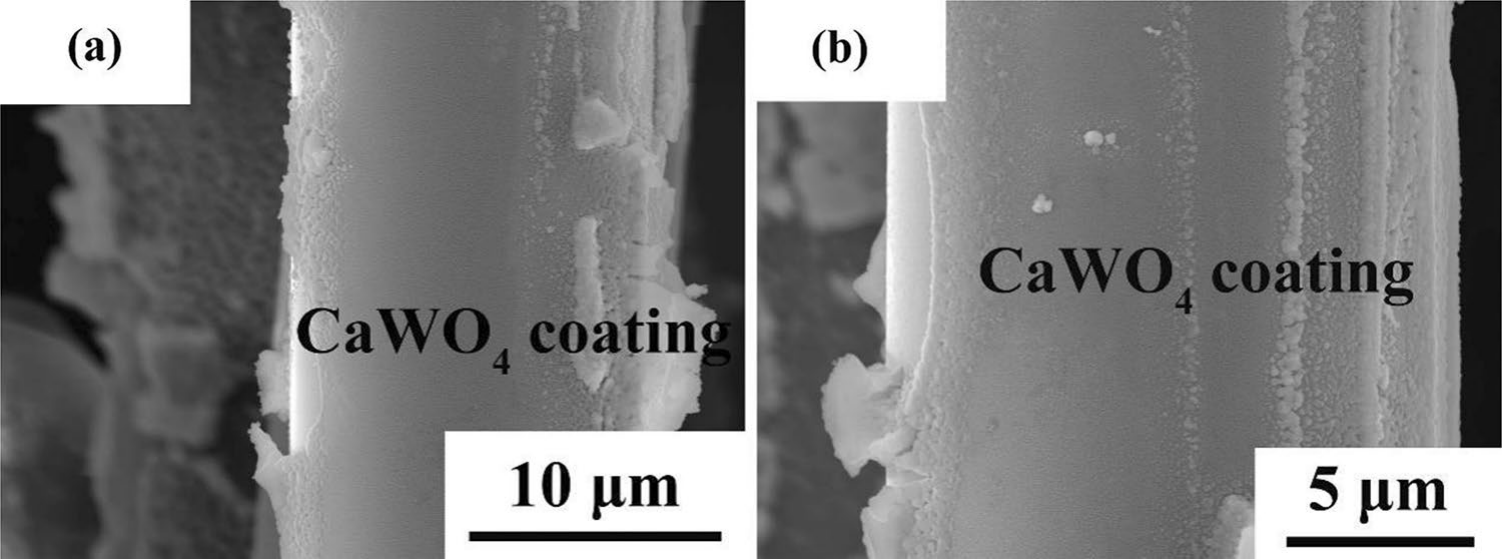
Surface morphology of CaWO_4_ coated SiC fiber.

Figure 5 shows the surface morphology of the coated SiC fiber. Together with XRD, it can be confirmed that CaWO_4_ interphase coating was successfully prepared on SiC fibers. If compared to interphase coating fabricated by CVD methods, e.g. BN coating in^7^, the CaWO_4_ interphase coating prepared by the so-gel method is relatively rougher.

**Figure 6:**
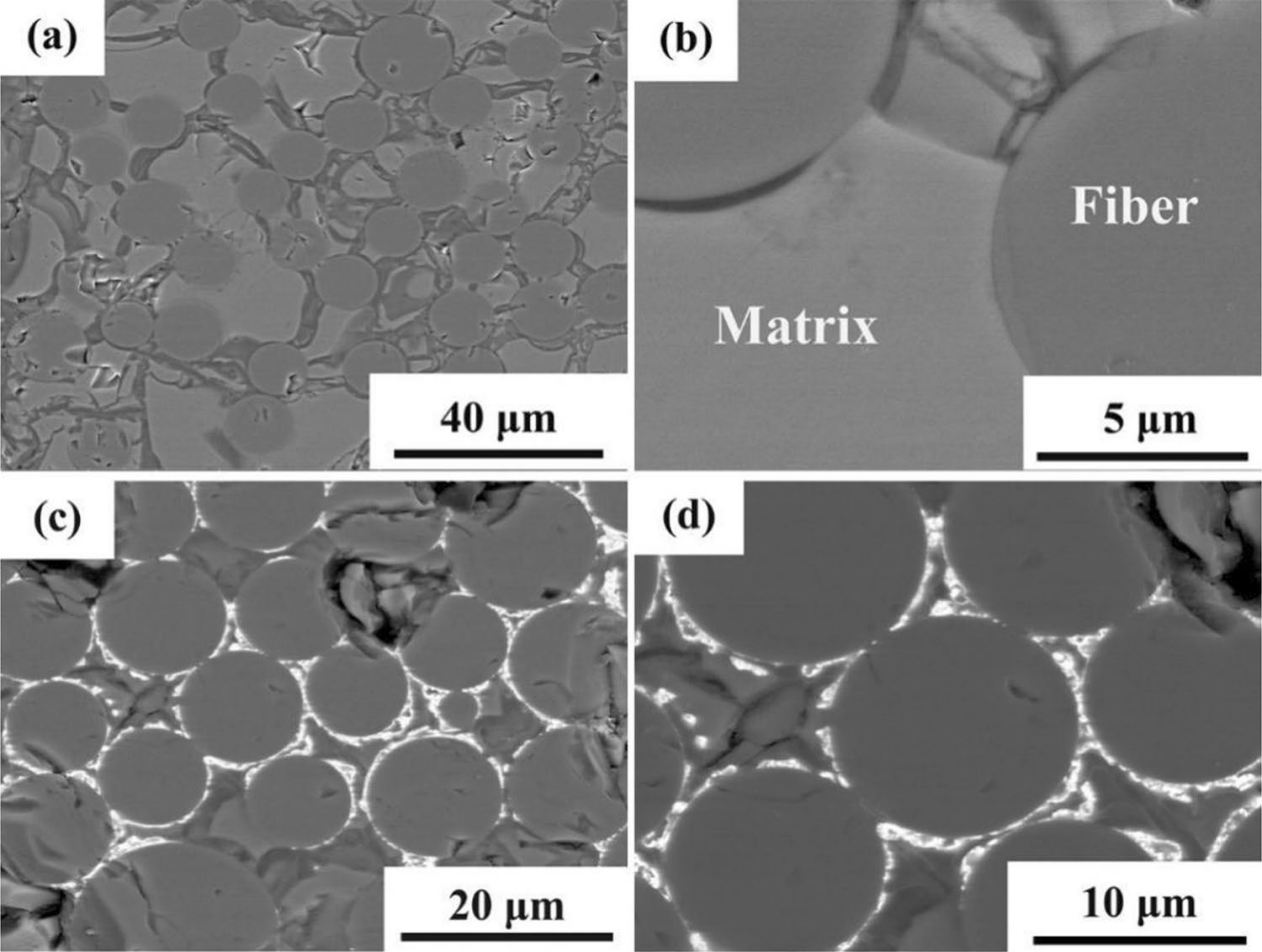
Cross-section view of SiC_f_/SiC minicomposites: (**a**,**b**) without interphase coating and (**c**,**d**) with CaWO_4_ interphase coating.

The size of the unidirectional SiC_f_/SiC minicomposite prepared by the PIP method is about 2 × 3 × 20 mm. The cross-section view of SiC_f_/SiC minicomposites (SEM backscattered electron images) with and without the CaWO_4_ interphase coating is demonstrated in Fig. 6. As shown in Figs. 5, 6, there exist regions around the fiber with no interphase coating. Mud cracks often observed in composites prepared by the PIP method^26^ were also found in the current SiC_f_/SiC minicomposites as shown in Fig. 6. According to Fig. 6c,d, in the coated area the CaWO_4_ interphase coatings have a thickness in the range of 200–600 nm.

The density of SiC_f_/SiC minicomposites with and without the CaWO_4_ interphase coating is about 2.16 g/cm^3^ and 2.29 g/cm^3^, respectively, and their porosity is about 14.4% and 10% respectively. The higher porosity of SiC_f_/SiC composites with the CaWO_4_ interfacial coating is probably due to its influence on the impregnation efficiency of PCS. The distribution of CaWO_4_ interphase coating prepared through sol–gel method is relatively random and liable to aggregate on the fiber surface, which may obstruct the impregnation of PCS. The lower impregnation efficiency of PCS can cause less SiC matrix filled inside the fiber tow, which leads to the higher porosity of the SiC_f_/SiC minicomposite.

### Interfacial property of minicomposites

**Figure 7:**
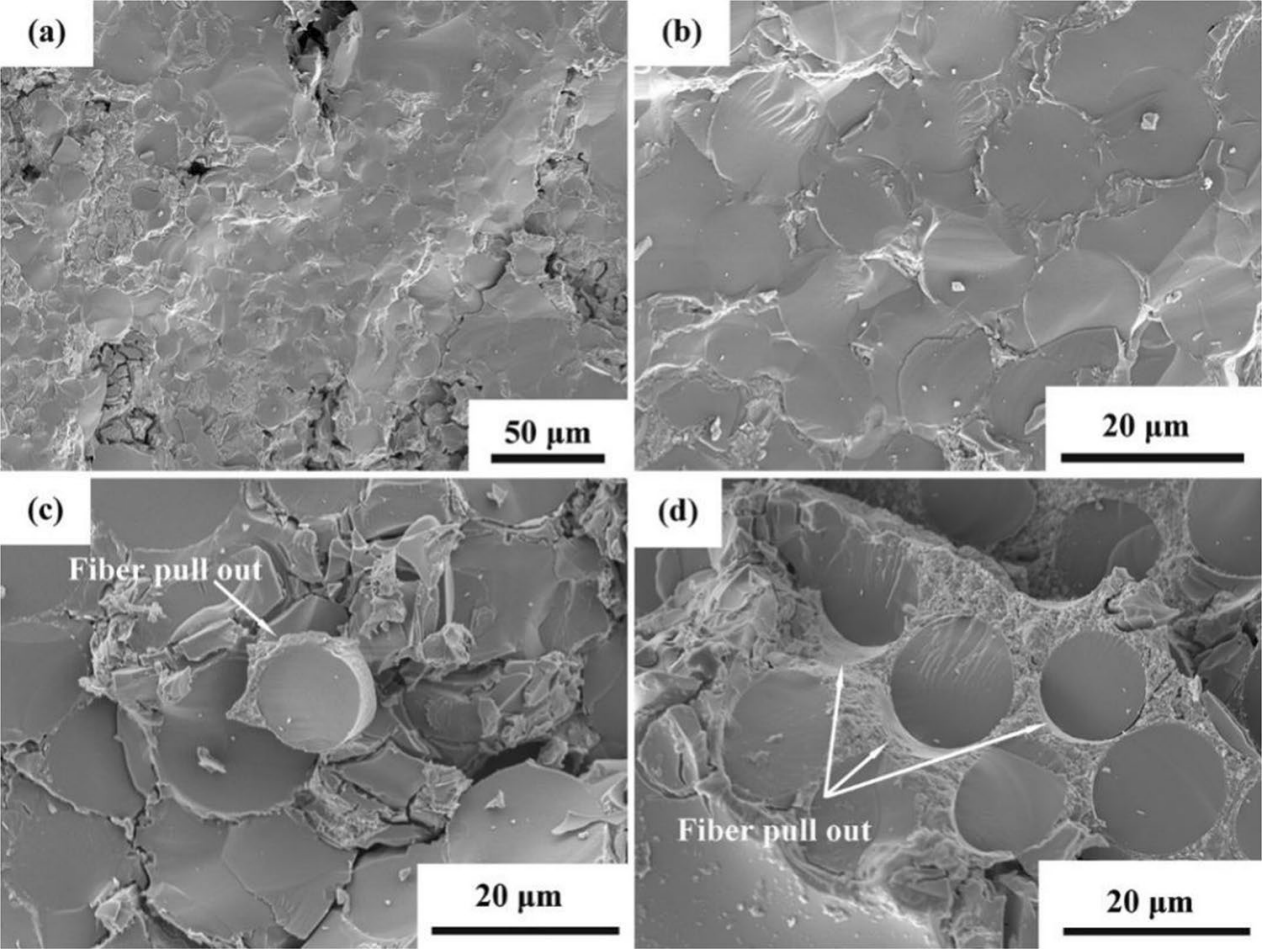
High-magnification fracture surface of SiC_f_/SiC minicomposites: (**a**,**b**) without interphase coating and (**c**,**d**) with CaWO_4_ interphase coating.

As one of the main functions of the interphase coating is to render pseudoplasticity to the composites, it is necessary to study the fracture behavior of the interphase-modified composite. As the prepared minicomposite samples were too small for carrying out reliable macroscopic tensile tests, the effect of the CaWO_4_ interphase coating on fracture behavior of SiC_f_/SiC minicomposites was mainly investigated by comparing the SEM fractography of the composite with and without the interphase. Fracture surfaces of the minicomposites are demonstrated in Fig. 7. The fracture surfaces of the minicomposite were generated through the in-situ tensile test as described in the experimental section. Flat fracture surfaces were obtained for the minicomposite without interphase coatings, and indications of crack deflection at the fiber/matrix interface or fiber pullout were not found (Fig. 7a,b). The SEM examination of the minicomposite without the interphase coating suggests clearly a brittle failure.

In the case of minicomposites reinforced with CaWO_4_ coated fibers, a rugged fracture surface was found. Debonding was observed to occur at the fiber/matrix interface, and distinct fiber pullouts were found on the fracture surface (Fig. 7c). In addition, SEM observation showed trough formation in the matrix after fiber pullout (Fig. 7d).

By comparing the fracture surface of SiC_f_/SiC minicomposites with and without CaWO_4_ interphase coating, it is indicated that CaWO_4_ interphase coating has a positive effect in changing the fracture behavior from a brittle manner to a more ductile one. When no interphase coating is present at the fiber/matrix interface, a strong bonding is formed, and as a result, cracks penetrate the fibers without any deflection during the failure of composites^27–29^. Hence, a flat fracture surface was obtained suggesting minimal fracture energy. In the SiC_f_/CaWO_4_/SiC minicomposite, the existence of CaWO_4_ interphase coating can provide a weaker interface bonding promoting the fiber/matrix interface debonding and fiber pullout during failure. Consequently, a rugged fracture surface was formed associated with the consumption of higher fracture energies.

**Figure 8:**
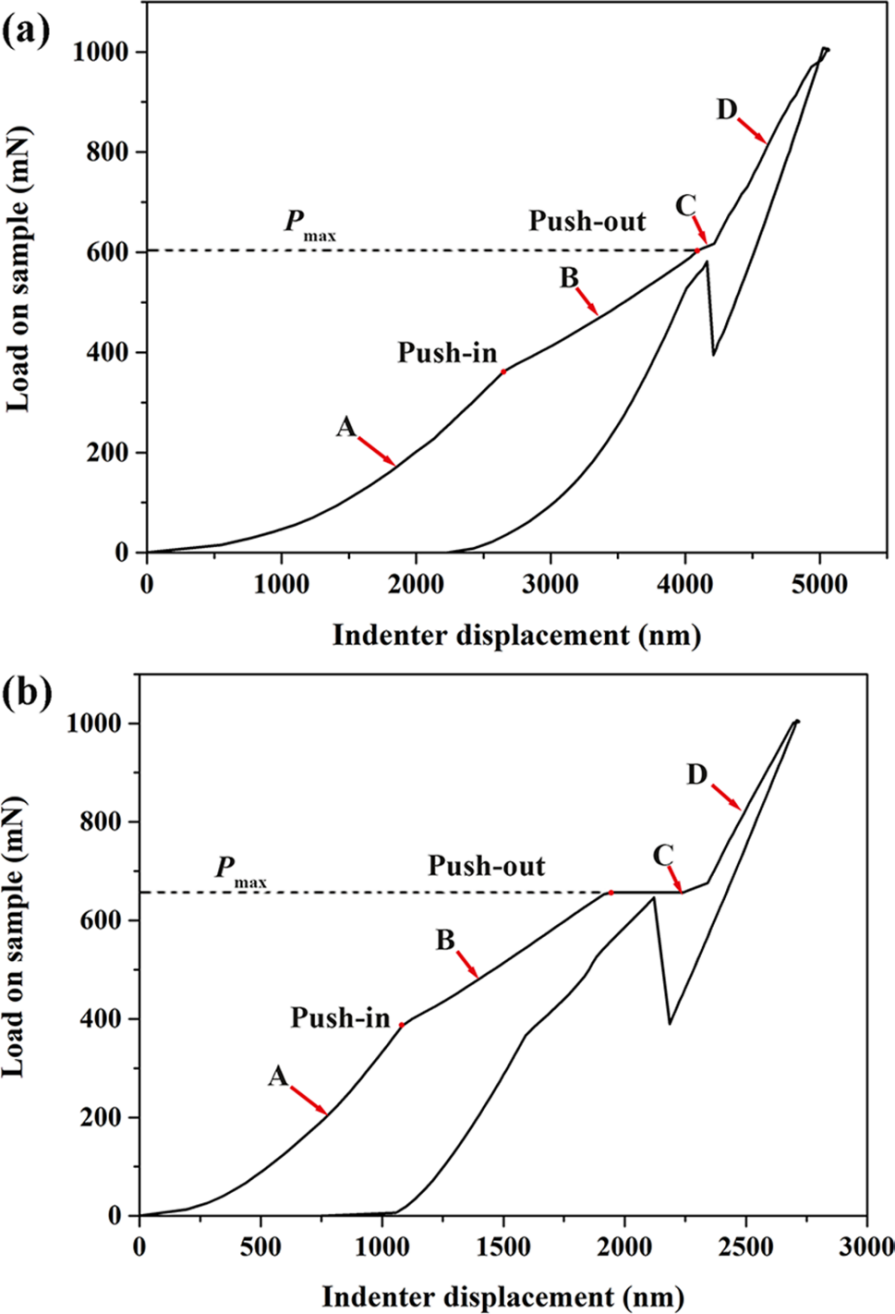
Representative indentation curve of single-fiber push-out tests on the SiC_f_/SiC minicomposite: (**a**) with CaWO_4_ interphase coating and (**b**) without interphase coating.

**Figure 9:**
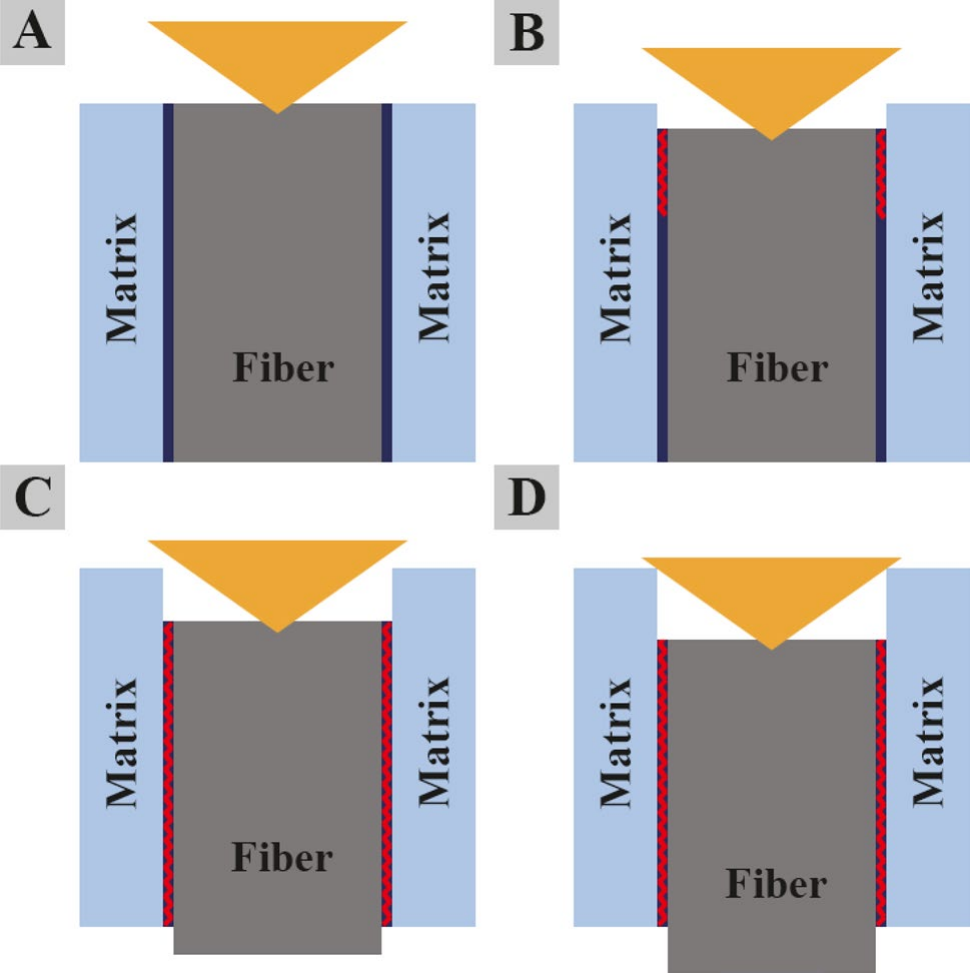
Schematic diagram of the fiber push-out process loaded by nanoindentation performed on the SiC_f_/CaWO_4_/SiC minicomposite. Adapted from reference^25^.

The effect of the CaWO_4_ coating on the interfacial property of the minicomposites is further evaluated quantitatively through the fiber push-out test. Fibers located above the groove and surrounded by the matrix were chosen randomly for push-out tests. To facilitate the push-out experiment accounting for the potentially stronger fiber/matrix bonding, the SiC_f_/SiC minicomposites without interphase coating were ground and polished to a thinner slice allowing the use of lower push-out loads. Figure 8 shows the typical indentation curve of SiC_f_/SiC minicomposites with (Fig. 8a) and without interphase coating (Fig. 8b) obtained from the single fiber push-out test. A similar single fiber push-out curve was reported by Zhang et al. ^25^. According to the load–displacement curve, the push-out process of a single fiber in the SiC_f_/CaWO_4_/SiC minicomposite can be divided into four periods (A, B, C, and D) which were marked in the indentation curve (Fig. 8). Figure 9 indicates a schematic diagram of these four periods in the fiber push-out process^25^.

In period A, the indenter presses into the SiC fiber, and the elastic deformation of SiC fiber occurs. The fiber/matrix interface is still in good condition with no cracks formed at the interface. During this period, the impact energy of the indenter is entirely assimilated by the elastic deformation of the SiC fiber^25^. In period B, partial debonding occurs at the fiber/matrix interface, and small cracks are formed at the interface caused by the decoupling of fiber and matrix. As a result, Young’s modulus of the fiber/matrix system decreases as shown in Fig. 8. In period C, the fiber/matrix interface is entirely debonded. The fiber push-out is initiated and then SiC fiber is pushed below the surface of the slice. The critical load when SiC fiber is pushed out is denoted as, *P*_*max*_ (Fig. 8). Finally, in period D, the indenter touches the surrounding SiC matrix causing the increase of Young’s modulus of the system.

The interfacial debonding stress of SiC_f_/CaWO_4_/SiC minicomposite can be calculated as follows^30, 31^

1 $$\tau = \frac{{P_{max} }}{\pi DL}$$

where *τ* is the interfacial debonding stress, *P*_*max*_ the push-out load, *D* the diameter of SiC fiber, and *L* the thickness of the slice.

About 15 push-out tests were carried out for the minicomposite. The average interfacial debonding stress of SiC_f_/SiC minicomposite with CaWO_4_ interphase coating and without interphase coating is calculated to be 80.7 ± 4.6 and 130.7 ± 0.1 MPa respectively, using Eq. (1). The interfacial debonding stress of composites with BN and PyC coating is about 5–20 MPa^32, 33^, and the debonding stress of composites with LaPO_4_ interphase is about 100–200 MPa^34, 35^. Lower interfacial debonding stress represents a weaker interface bonding and facilitates the toughening of the composites. Hence, the effectiveness of CaWO_4_ interphase for toughening is likely to be worse than BN or PyC coating but slightly better or close to the LaPO_4_ coating.

Based on the fracture surface investigation and interfacial debonding stress measurements, it is evident that the CaWO_4_ interphase indeed improved the interfacial property by providing a weaker bonding at the orginal fiber/matrix interface. It is noted, however, that the toughnening effect is not dramastic in the current work as indicated by the limited debonding length and matrix cracking observed from the fracture surface. This should be at least partly due to the localized incompleteness of the coating as seen in Fig. 6. It is believed that future optimization of the coating process would enable a complete utilization of the toughening effect provided by the CaWO_4_ interphase.

### Effect of oxidation on the interphase and composite

**Figure 10:**
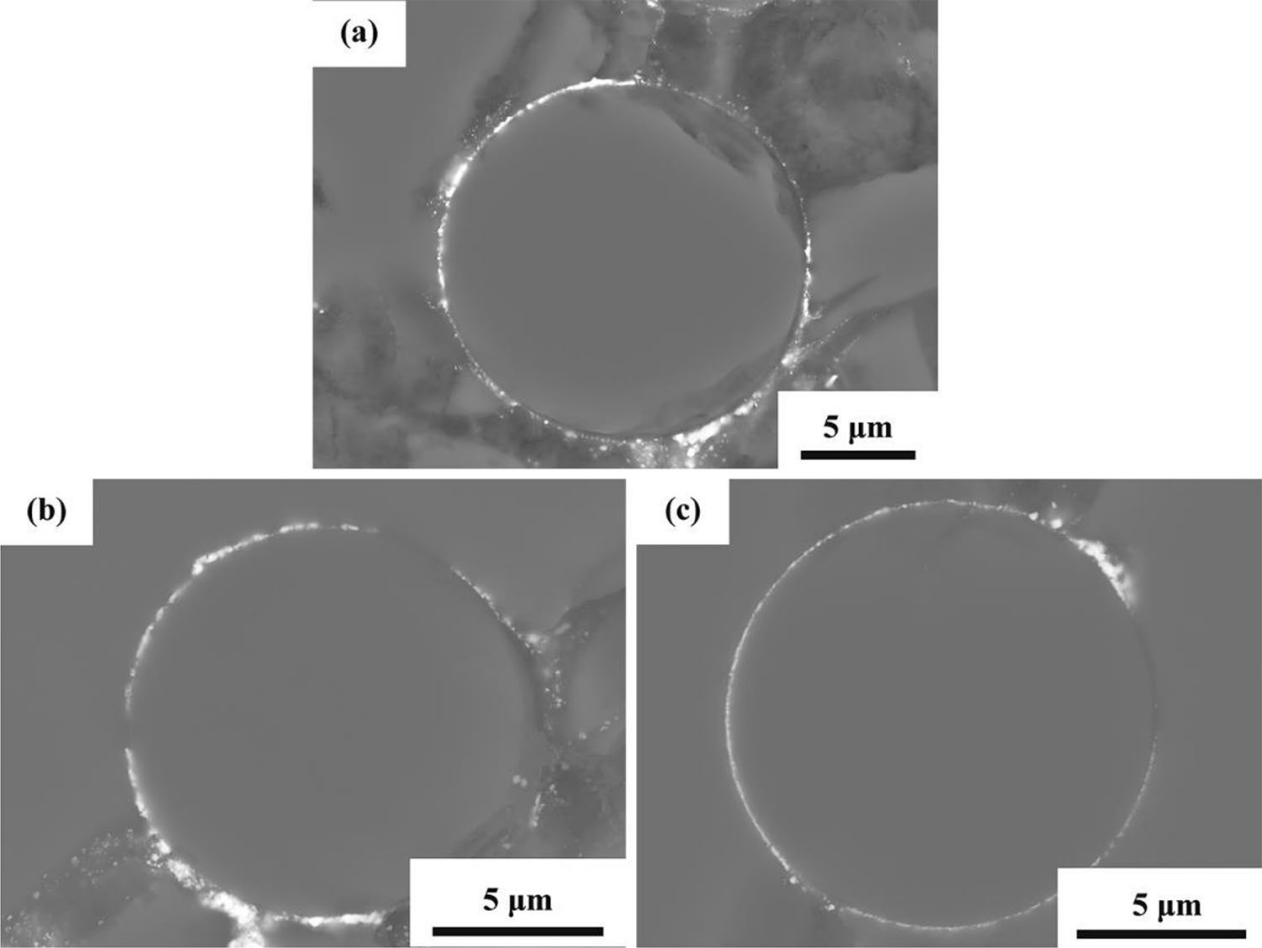
Cross-section view of SiC_f_/CaWO_4_/SiC minicomposites oxidized at (**a**) 1000 °C for 50 h, (**b**) 1100 °C for 10 h, and (**c**) 1100 °C for 50 h.

The SiC_f_/CaWO_4_/SiC minicomposite was oxidized at 1000 °C for 50 h and 1100 °C for 10 h and 50 h, and the cross-section view of the oxidized minicomposites is demonstrated in Fig. 10. According to the SEM observation, the CaWO_4_ interphase coating was still visible in the composite after the oxidation process, confirming that the CaWO_4_ interphase coating has a good oxidation resistance as expected. Hence, it can be speculated that CaWO_4_ interphase coating would be able to promote crack deflection and fiber pullout in the oxygen-rich environment for a long time.

**Figure 11:**
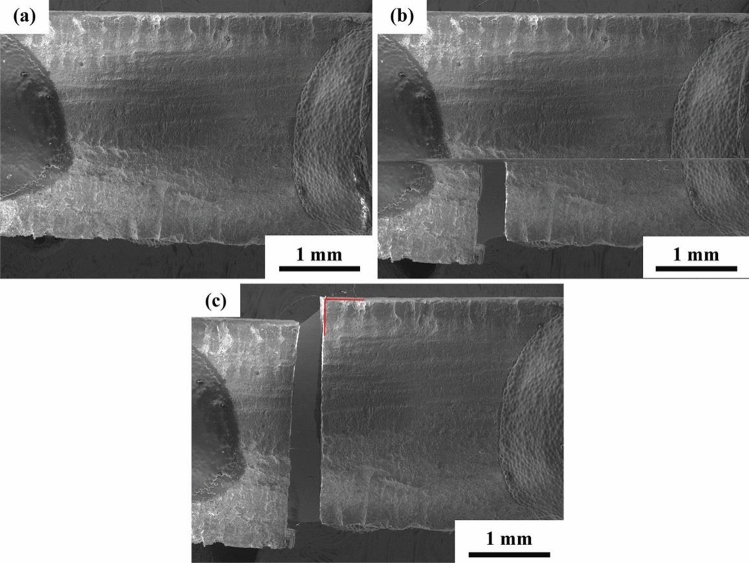
In-situ tensile SEM observation of SiC_f_/SiC minicomposite without interphase coatings.

**Figure 12:**
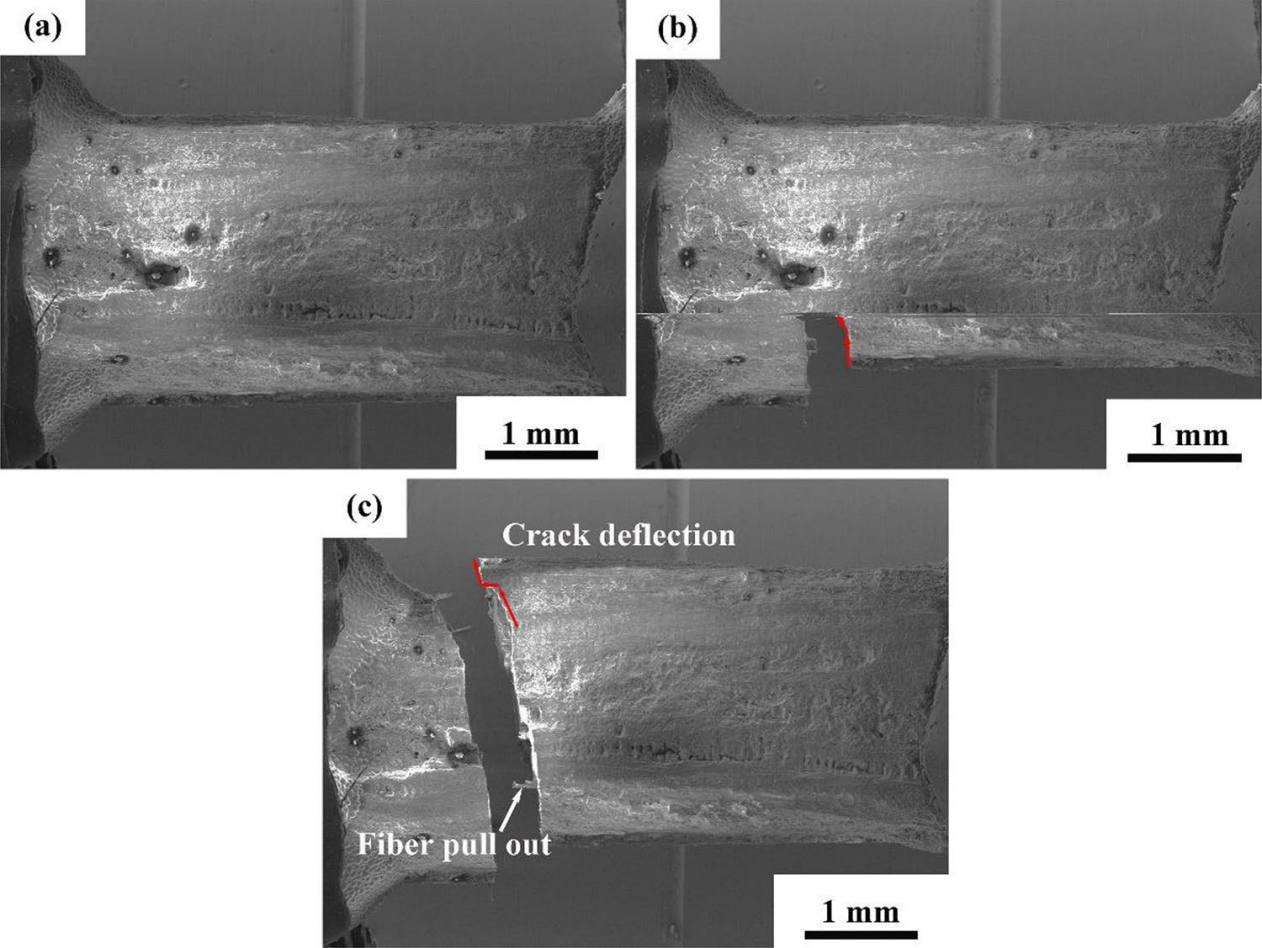
In-situ tensile SEM observation of SiC_f_/CaWO_4_/SiC minicomposite oxidized at 1100℃ for 50 h.

In-situ tensile SEM observation of the SiC_f_/CaWO_4_/SiC minicomposite after being oxidized at 1100 °C for 50 h was carried out to evaluate the effectiveness of CaWO_4_ interphase coating after oxidation. Unoxidized SiC_f_/SiC minicomposite without the interphase coating was also investigated for comparison. SEM micrographs obtained in-situ during the tensile testing of the SiC_f_/SiC and oxidized SiC_f_/CaWO_4_/SiC minicomposite are shown in Figs. 11, 12, respectively. The entire failure process of SiC_f_/SiC minicomposite was completed within less than 2 s. It is obvious that the crack propagation path of SiC_f_/SiC minicomposite is almost perpendicular to the upper and lower surfaces of the composite, and the crack penetrates the fibers without any deflection (Fig. 11). The flat fracture surface of SiC_f_/SiC minicomposite is in accordance with the fracture surface morphology shown in Fig. 6.

**Figure 13:**
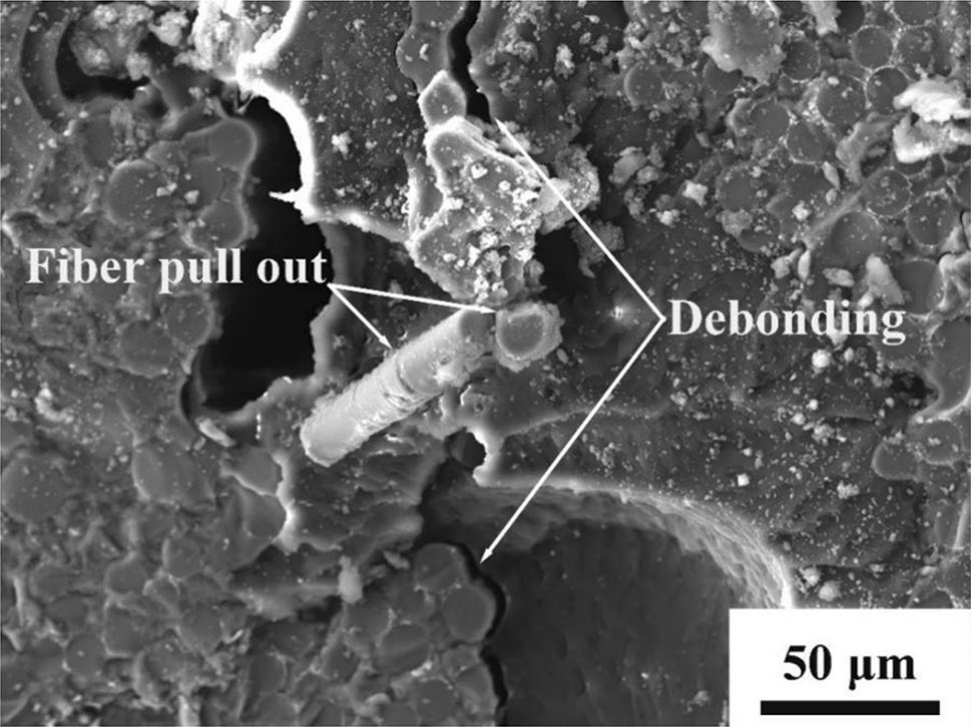
Fractography of SiC_f_/CaWO_4_/SiC minicomposite oxidized at 1100 °C for 50 h.

In comparison, crack deflection and apparent fiber pullout were indicated during the failure of SiC_f_/CaWO_4_/SiC minicomposite that has been oxidized at 1100 °C for 50 h (Fig. 12). Figure 13 further shows the fractography of SiC_f_/CaWO_4_/SiC minicomposite oxidized at 1100 °C for 50 h. Fiber pullout and fiber/matrix interface debonding were observed on the fracture surface which confirms that the CaWO_4_ interphase coating remains effective in toughening the minicomposite after oxidation. This is in high contrast to the PyC and BN coating, which are prone to be consumed by oxidation at intermediate temperatures leading to strong bonding at the fiber/matrix interface and the loss of crack deflection functionality^36, 37^.

### Thermal compatibility between CaWO_4_ and SiC

**Figure 14:**
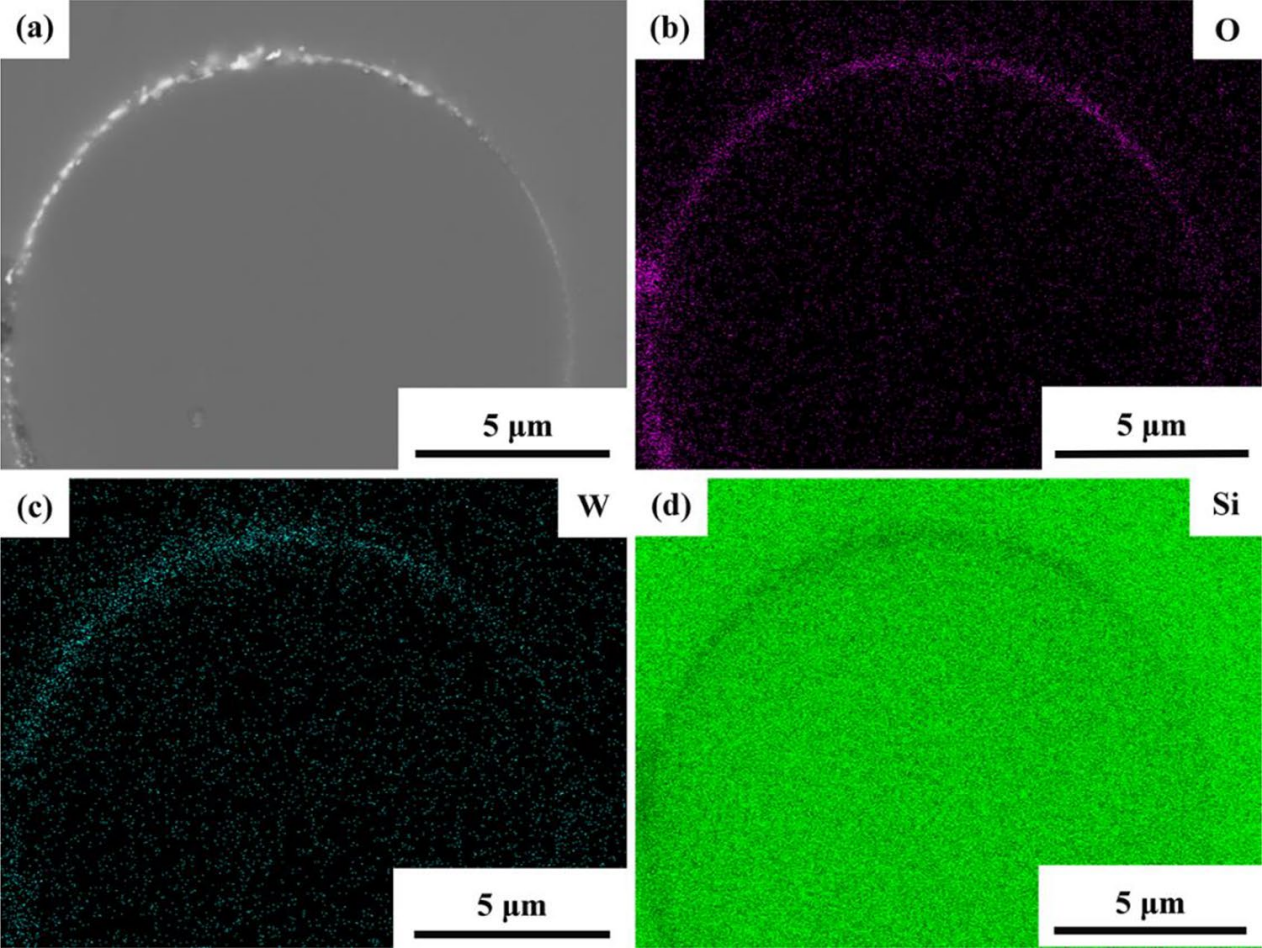
(**a**) Cross-section view of SiC_f_/CaWO_4_/SiC minicomposite heat-treated at 1300 °C for 50 h. (**b**–**d**) Corresponding EDS map scanning results.

The SiC_f_/CaWO_4_/SiC minicomposite was heat-treated at a higher temperature of 1300 °C for 50 h to evaluate the thermal compatibility between CaWO_4_ and SiC. The cross-section image and corresponding EDS map from the interface area are shown in Fig. 14. The CaWO_4_ interphase coating remains visible at the fiber/matrix interface and no obvious reaction zone is found between interphase and fiber or matrix. Meanwhile, the EDS mapping indicates the absence of Si in the interphase and W or O in the SiC matrix or fiber, which further confirms that no interfacial reaction happens after the heat treatment at 1300 °C for 50 h. Compared with the LaPO_4_ coating that has been shown reacting with SiC at 1200 °C^15^, the good thermal compatibility between CaWO_4_ interphase and SiC at 1300 °C suggests that CaWO_4_ interphase is more suitable for SiC_f_/SiC composites.

## Conclusions

Relatively uniform CaWO_4_ interphase coatings were prepared on the SiC fiber using the sol–gel dip-coating method and unidirectional SiC_f_/CaWO_4_/SiC minicomposites were then fabricated through the PIP method. The thickness of the interphase coating was in the range of 200–600 nm. The interfacial property of SiC_f_/CaWO_4_/SiC minicomposite was quantitatively evaluated and the thermal stability of CaWO_4_ interphase coating at 1000–1300 °C in air was investigated. The results are summarized as:

1. Crack deflection and fiber pullout are observed during the failure of the SiC_f_/CaWO_4_/SiC minicomposite, indicating that a weak fiber/matrix interface is provided by CaWO_4_ interphase coating. The interfacial debonding stress of SiC_f_/SiC minicomposite with the CaWO_4_ interphase coating is about 80.7 ± 4.6 MPa which is between that of PyC and LaPO_4_ coating.
2. Different from PyC and BN coating, the CaWO4 interphase coating is confirmed to have a good oxidation resistance. The CaWO_4_ interphase coating is preserved at the interface and can promote crack deflection and fiber pullout after the composite is oxidation at 1000–1100 °C for 10–50 h.
3. The CaWO_4_ interphase coating has no obvious interfacial reaction and diffusion with SiC fiber or matrix after heat treatment at 1300 °C for 50 h, suggesting its better thermal compatibility with SiC than the LaPO_4_ coating. Combined with the suitable interphase bonding stress, the CaWO_4_ interphase coating has a good potential to be used in SiC_f_/SiC composites.

## Data Availability

Datasets used or analyzed during the current study are available from the corresponding author on a reasonable request.
